# Supplementary material for: A Small-Scale Analysis of Elevational Species Richness and Beta Diversity Patterns of Arthropods on an Oceanic Island (Terceira, Azores)
Source: Insects. 2021 Oct 14;12(10):936. doi: 10.3390/insects12100936 (PMC8538719; doi:10.3390/insects12100936)
Supplement: Supplementary file 1 [file insects-12-00936-s001.zip › insects-1420874-supplementary.pdf]

Supporting Information

A small-scale analysis of elevational species richness and beta diversity patterns of arthropods in an oceanic island (Terceira, Azores)

Content:

Table S1: Transect species

Table S2: Sample coverage

Table S3: Alpha-diversity measures

Table S4: Alpha-diversity linear regression coefficients

Table S5: Relative dominance of alpha- and beta-diversity

Table S6: Beta-diversity measures

Table S7: Beta-diversity MRM coefficients

Table S8: Beta-diversity replacement or richness dominance

Figure S1. Image of a SLAM (Sea, Land, and Air Malaise) trap

Figure S2: All sites alpha-diversity patterns (observed, estimated total & mean species richness)

Figure S3: Transect alpha-diversity patterns (estimated total & mean species richness, and alpha-diversity patterns for only indigenous species of each order)

Figure S4: Transect Hill numbers *q*2 – *q*4 (exponential Shannon, inverse Simpson & Berger-Parker)

Figure S5: Beta-diversity patterns over elevational distance for the four different orders

Figure S6: Beta-diversity patterns over elevational distance for native, endemic and introduced species

Table S1. List of arthropod species recorded and identified along the five-site elevation transect presented here (summer records only). The numbers correspond to Figure 2 (All species). Column 3 shows the status of each species (N = native non-endemic, E = endemic, I = introduced); columns 4-5 show for each species the abundance-based mean elevation and the elevational range of its occurrence; column 6 shows the number of specimens that was recorded along the whole transect per species.

	Species	Order	Status	Elevation [m a.s.l.] mean	Elevation [m a.s.l.] range	N
1	*Pseudophloeophagus truncorum* (Stephens, 1831)	Coleoptera	N	46	46	1
2	*Psylliodes marcida* (Illiger, 1807)	Coleoptera	N	46	46	2
3	*Scymnus interruptus* (Goeze, 1777) + *Scymnus nubilus* Mulsant, 1850	Coleoptera	N	46	46	1
4	*Stilbus testaceus* (Panzer, 1797)	Coleoptera	N	46	46	1
5	*Oligota pumilio* Kiesenwetter, 1858	Coleoptera	N	46	46	1
6	*Scolopostethus decoratus* (Hahn, 1833)	Hemiptera	N	46	46	38
7	*Kelisia ribauti* Wagner, 1938	Hemiptera	N	46	46	1
8	*Loricula coleoptrata* (Fallén, 1807)	Hemiptera	N	46	46	1
9	*Muellerianella* sp.3	Hemiptera	N	46	46	1
10	*Emblethis denticollis* Horváth, 1878	Hemiptera	N	46	46	1
11	*Beosus maritimus* (Scopoli, 1763)	Hemiptera	N	46	46	1
12	*Plinthisus minutissimus* Fieber, 1864	Hemiptera	N	46	46	5293
13	*Monomorium carbonarium* (Smith, 1858)	Hymenoptera	N	46	46	79
14	*Temnothorax unifasciatus* (Latreille, 1798)	Hymenoptera	N	46	46	33
15	*Tetramorium caespitum* (Linnaeus, 1758)	Hymenoptera	N	46	46	1
16	*Ectopsocus strauchi* Enderlein, 1906	Psocoptera	N	46	46	61
17	*Aeolothrips gloriosus* Bagnall, 1914	Thysanoptera	N	46	46	2
18	*Neon acoreensis* Wunderlich, 2008	Araneae	E	46	46	1
19	*Nysius atlantidum* Horváth, 1890	Hemiptera	E	46	46	1
20	*Pseudeuophrys vafra* (Blackwall, 1867)	Araneae	I	46	46	3
21	*Salticus mutabilis* Lucas, 1846	Araneae	I	46	46	3
22	*Coccotrypes carpophagus* (Hornung, 1842)	Coleoptera	I	46	46	3
23	*Atheta fungi* (Gravenhorst, 1806)	Coleoptera	I	46	46	1
24	*Amara aenea* (DeGeer, 1774)	Coleoptera	I	46	46	1
25	*Aeolus melliculus moreleti* Tarnier, 1860	Coleoptera	I	46	46	1
26	*Melanotus dichrous* (Erichson, 1841)	Coleoptera	I	46	46	3
27	*Longitarsus kutscherai* (Rye, 1872)	Coleoptera	I	46	46	2
28	*Clitostethus arcuatus* (Rossi, 1794)	Coleoptera	I	46	46	1
29	*Coproporus pulchellus* (Erichson, 1839)	Coleoptera	I	46	46	1
30	*Aleochara clavicornis* L. Redtenbacher, 1849	Coleoptera	I	46	46	1
31	Gen. sp.3	Coleoptera	I	46	46	3
32	Gen. sp.2	Hemiptera	I	46	46	1
33	Gen. sp.	Psocoptera	I	46	46	5
34	*Scutigera coleoptrata* (Linnaeus, 1758)	Scutigeromorpha	I	46	46	127
35	*Hercinothrips bicinctus* (Bagnall, 1919)	Thysanoptera	I	46	46	4
36	Gen. sp.	Hemiptera	N?	46	46	2
37	*Pilophorus confusus* (Kirschbaum, 1856)	Hemiptera	N	58	46-231	15

38	Ephippiochthonius tetrachelatus (Preyssler, 1790)	Pseudoscorpiones	I	67	46-231	9
39	Gen. sp.4	Hemiptera	I	67	46-404	62
40	*Heliothrips haemorrhoidalis* (Bouché, 1833)	Thysanoptera	I	72	46-404	110
41	*Heteroderes azoricus* (Tarnier, 1860)	Coleoptera	E	77	46-231	6
42	*Dilta saxicola* (Womersley, 1930)	Microcoryphia	N	81	46-404	61
43	*Strophingia harteni* Hodkinson, 1981	Hemiptera	E	82	46-693	158
44	*Macaroeris diligens* (Blackwall, 1867)	Araneae	N	83	46-231	5
45	*Kleidocerys ericae* (Horváth, 1909)	Hemiptera	N	93	46-404	941
46	*Trichopsocus clarus* (Banks, 1908)	Psocoptera	N	112	46-693	84
47	*Bertkauia lucifuga* (Rambur, 1842)	Psocoptera	N	114	46-404	24
48	*Sericoderus lateralis* (Gyllenhal, 1827)	Coleoptera	I	129	46-404	20
49	*Lasius grandis* Forel, 1909	Hymenoptera	N	130	46-404	175
50	*Eupteryx filicum* (Newman, 1853)	Hemiptera	N	136	46-404	4
51	*Ommatoiulus moreleti* (Lucas, 1860)	Julida	I	147	46-404	32
52	Gen. sp.2	Psocoptera	I	168	46-404	9
53	*Aspidapion radiolus* (Marsham, 1802)	Coleoptera	N	169	46-231	6
54	*Anaspis* sp.	Coleoptera	I	174	46-404	10
55	*Tetramorium* sp.	Hymenoptera	I	175	46-693	5
56	*Loricula elegantula* (Bärensprung, 1858)	Hemiptera	N	187	46-404	17
57	*Elipsocus azoricus* Meinander, 1975	Psocoptera	E	225	46-404	77
58	*Cryptophagus* sp.1	Coleoptera	I	227	46-404	3
59	*Valenzuela burmeisteri* (Brauer, 1876)	Psocoptera	N	230	46-404	160
60	*Tenuiphantes miguelensis* (Wunderlich, 1992)	Araneae	N	231	231	1
61	*Xysticus cor* Canestrini, 1873	Araneae	N	231	231	1
62	*Cryptolestes* sp.1	Coleoptera	N	231	231	2
63	*Campyloneura virgula* (Herrich-Schaeffer, 1835)	Hemiptera	N	231	231	8
64	*Valenzuela burmeisteri* (Brauer, 1876)	Psocoptera	N	231	231	1
65	*Parasteatoda tepidariorum* (C. L. Koch, 1841)	Araneae	I	231	231	1
66	*Textrix caudata* L. Koch, 1872	Araneae	I	231	231	9
67	*Steatoda grossa* (C. L. Koch, 1838)	Araneae	I	231	231	1
68	*Cryptachaea blattea* (Urquhart, 1886)	Araneae	I	231	231	8
69	*Entelecara schmitzi* Kulczynski, 1905	Araneae	I	231	231	2
70	*Steatoda nobilis* (Thorell, 1875)	Araneae	I	231	231	1
71	*Dromius meridionalis* Dejean, 1825	Coleoptera	I	231	231	1
72	*Cryptamorpha desjardinsii* (Guérin-Méneville, 1844)	Coleoptera	I	231	231	1
73	Gen. sp.5	Coleoptera	I	231	231	1
74	*Epitrix cucumeris* (Harris, 1851)	Coleoptera	I	231	231	1
75	*Sitona discoideus* Gyllenhal, 1834	Coleoptera	I	231	231	2
76	*Popillia japonica* Newman, 1838	Coleoptera	I	231	231	1
77	*Acizzia uncatoides* (Ferris & Klyver, 1932)	Hemiptera	I	231	231	1
78	Gen. sp.3	Psocoptera	I	231	231	1
79	*Xyleborinus alni* Nijima, 1909	Coleoptera	I	235	231-404	48
80	*Ectopsocus briggsi* McLachlan, 1899	Psocoptera	I	267	46-693	14
81	*Tenuiphantes tenuis* (Blackwall, 1852)	Araneae	I	288	46-404	21
82	*Anaspis proteus* Wollaston, 1854	Coleoptera	N	319	46-693	41

83	*Anobium punctatum* (De Geer, 1774)	Coleoptera	I	337	46-404	45
84	*Atlantopsocus adustus* (Hagen, 1865)	Psocoptera	N	348	46-404	31
85	*Porrhoclubiona decora* (Blackwall, 1859)	Araneae	N	349	46-404	26
86	*Valenzuela flavidus* (Stephens, 1836)	Psocoptera	N	361	46-930	76
87	*Monalocoris filicis* (Linnaeus, 1758)	Hemiptera	N	370	46-693	2
88	*Lasaeola oceanica* Simon, 1883	Araneae	E	370	46-693	2
89	*Theridion musivivum* Schmidt, 1956	Araneae	N	404	404	1
90	*Proteinus atomarius* Erichson, 1840	Coleoptera	N	404	404	1
91	*Piezodorus lituratus* (Fabricius, 1794)	Hemiptera	N	404	404	2
92	*Agalenatea redii* (Scopoli, 1763)	Araneae	I	404	404	1
93	*Pelecopsis parallela* (Wider, 1834)	Araneae	I	404	404	2
94	*Nigma puella* (Simon, 1870)	Araneae	I	404	404	1
95	*Cordalia obscura* (Gravenhorst, 1802)	Coleoptera	I	404	404	1
96	*Leiobunum blackwalli* Meade, 1861	Opiliones	N	406	46-693	44
97	*Zetha simonyi* (Krauss, 1892)	Blattodea	N	425	404-693	28
98	*Drouetius borgesi borgesi* (Machado, 2009)	Coleoptera	E	466	404-693	37
99	*Tachyporus chrysomelinus* (Linnaeus, 1758)	Coleoptera	I	488	46-930	2
100	*Pseudophloeophagus tenax* Wollaston, 1854	Coleoptera	N	490	231-693	43
101	*Metellina merianae* (Scopoli, 1763)	Araneae	I	500	404-693	3
102	*Cyphopterum adcendens* (Herrich-Schäffer 1835)	Hemiptera	N	528	46-693	49
103	*Pinalitus oromii* J. Ribes 1992	Hemiptera	E	545	46-930	60
104	*Cixius azoterceirae* Remane & Asche, 1979	Hemiptera	E	561	46-930	698
105	*Lithobius pilicornis pilicornis* Newport, 1844	Lithobiomorpha	N	571	46-693	10
106	*Tachyporus nitidulus* (Fabricius, 1781)	Coleoptera	I	581	231-930	2
107	*Hemerobius azoricus* Tjeder, 1948	Neuroptera	E	586	46-930	24
108	*Gibbaranea occidentalis* Wunderlich, 1989	Araneae	E	597	404-693	6
109	*Microlinyphia johnsoni* (Blackwall, 1859)	Araneae	N	635	404-693	5
110	*Trioza laurisilvae* Hodkinson, 1990	Hemiptera	N	648	46-930	50
111	*Calacalles subcarinatus* (Israelson, 1984)	Coleoptera	E	669	404-693	12
112	*Trigoniophthalmus borgesi* Mendes, Gaju, Bach & Molero, 2000	Microcoryphia	E	676	404-693	17
113	*Macaroeris cata* (Blackwall, 1867)	Araneae	N	693	693	7
114	*Catops coracinus* Kellner, 1846	Coleoptera	N	693	693	1
115	*Brachysteles parvicornis* (A. Costa, 1847)	Hemiptera	N	693	693	1
116	*Canariphantes acoreensis* (Wunderlich, 1992)	Araneae	E	693	693	1
117	*Eupteryx azorica* Ribaut, 1941	Hemiptera	E	693	693	1
118	*Ero furcata* (Villers, 1789)	Araneae	I	693	693	7
119	*Cheiracanthium erraticum* (Walckenaer, 1802)	Araneae	I	693	693	1
120	*Chthonius ischnocheles* (Hermann, 1804)	Pseudoscorpiones	I	693	693	6
121	*Elipsocus brincki* Badonnel, 1963	Psocoptera	E	696	46-930	120
122	Gen. sp.	Hemiptera	E	704	46-930	475
123	*Ceratothrips ericae* (Haliday, 1836)	Thysanoptera	N	736	46-930	7
124	*Sancus acoreensis* (Wunderlich, 1992)	Araneae	E	752	693-930	4
125	*Savigniorrhipis acoreensis* Wunderlich, 1992	Araneae	E	772	693-930	12
126	*Lathys dentichelis* (Simon, 1883)	Araneae	N	821	404-930	7
127	*Acorigone acoreensis* (Wunderlich, 1992)	Araneae	E	835	693-930	20
128	*Rugathodes acoreensis* Wunderlich, 1992	Araneae	E	851	693-930	21

129	*Notothecta dryochares* (Israelson, 1985)	Coleoptera	E	896	693-930	7
130	*Walckenaeria grandis* (Wunderlich, 1992)	Araneae	E	930	930	1
131	*Erigone atra* Blackwall, 1833	Araneae	I	930	930	1
132	Gen. sp.1	Coleoptera	I	930	930	1
133	*Cercyon haemorrhoidalis* (Fabricius, 1775)	Coleoptera	I	930	930	1
134	Gen. sp.6	Hemiptera	I	930	930	2

Table S2 Sampling coverage estimates for all species, four different orders and native, endemic and introduced species, for: a) The five sampling sites along the elevation transect (summer records of the years 2015-2018); and b) the five elevation bands (represented by mean elevation) that reflect all 12 sampling sites on Terceira island (records of each season from autumn 2014 to summer 2018, rarified to a common sampling level of 15 samples per band). Coverage estimates are derived with the R package iNEXT [62].

a) Transect sites

**Species group**	**Elevation [m a.s.l.]**					
	**46**	**231**	**404**	**693**	**930**	*mean*
All species	1.00	0.95	0.97	0.98	0.98	0.98
Araneae	0.90	0.83	0.88	0.91	0.92	0.89
Coleoptera	0.80	0.91	0.94	0.95	0.63	0.84
Hemiptera	1.00	0.99	0.98	1.00	1.00	0.99
Psocoptera	0.99	0.94	1.00	0.78	1.00	0.94
Native	1.00	0.99	0.98	0.96	1.00	0.99
Endemic	0.98	0.87	0.99	0.99	1.00	0.97
Introduced	0.98	0.88	0.89	0.84	0.33	0.78
mean	0.95	0.92	0.96	0.93	0.86	0.92

b) All 12 sites

**Species group**	**Elevation [m a.s.l.]**					
	**46**	**231**	**491**	**672**	**910**	*mean*
All species	1.00	0.96	0.98	0.99	0.98	0.98
Araneae	0.88	0.89	0.94	0.98	0.99	0.94
Coleoptera	0.92	0.89	0.89	0.93	0.87	0.90
Hemiptera	1.00	0.98	0.99	0.99	0.99	0.99
Psocoptera	1.00	0.99	1.00	0.99	1.00	1.00
Native	1.00	0.98	0.99	0.98	0.90	0.97
Endemic	1.00	0.99	1.00	1.00	1.00	1.00
Introduced	0.98	0.90	0.88	0.80	0.60	0.83
mean	0.97	0.95	0.96	0.96	0.92	0.95

Table S3 Alpha-diversity measures for all species, four different orders and native, endemic and introduced species, providing: a) Observed species richness (S), b) estimated total (Jacknife-1) species richness and c) mean richness per sample (q1) for the five elevation transect sites (also including selection of only indigenous species for each order); d) observed species richness (S), e) estimated total (Jacknife-1) species richness and f) mean richness per sample (q1) for the five bands (represented by mean elevation) that reflect all 12 sites; and Hill number estimates of g) exponential Shannon (q2), h) inverse Simpson (q3) and Berger-Parker (q4) for the five elevation transect sites. For all estimates of observed, total and mean richness in a) – f), standard deviations are given in brackets for each estimate.

a) Transect observed richness

Species group	Elevation [m a.s.l.]				
	**46**	**231**	**404**	**693**	**930**
All	79 (5.0)	52 (5.0)	49 (3.5)	40 (3.3)	21 (2.6)
Araneae	7 (1.0)	10 (2.2)	10 (2.2)	13 (1.6)	7 (1.7)
Coleoptera	23 (3.1)	17 (2.3)	11 (2.5)	6 (0.8)	5 (1.5)
Hemiptera	22 (3.5)	10 (2.0)	10 (1.2)	9 (1.9)	5 (0.8)
Psocoptera	11 (1.2)	11 (2.8)	9 (0.8)	4 (1.3)	2 (0.0)
Native	38 (3.3)	20 (3.1)	22 (2.2)	16 (1.9)	4 (1.0)
Endemic	11 (2.5)	7 (2.0)	11 (1.6)	18 (1.8)	11 (1.0)
Introduced	29 (2.9)	25 (3.3)	16 (2.3)	6 (1.8)	6 (1.9)
Araneae ind.	4 (1.0)	3 (1.1)	5 (1.5)	10 (1.1)	6 (1.4)
Coleoptera ind.	8 (1.5)	5 (1.1)	5 (0.8)	6 (0.8)	1 (0.0)
Hemiptera ind.	19 (2.9)	8 (1.5)	9 (0.8)	9 (1.9)	4 (0.4)
Psocoptera ind.	8 (1.1)	8 (2.2)	7 (0.6)	3 (1.0)	2 (0.0)

b) Transect estimated total richness

Species group	Elevation [m a.s.l.]				
	**46**	**231**	**404**	**693**	**930**
All	110 (5.3)	80 (14.8)	64 (1.2)	55 (5.3)	28 (2.9)
Araneae	9 (2.3)	15 (0.8)	15 (0.8)	18 (3.6)	10 (2.7)
Coleoptera	35 (1.2)	25 (4.3)	16 (1.4)	8 (0.9)	8 (0.7)
Hemiptera	31 (2.1)	15 (3.6)	12 (0.8)	12 (2.1)	6 (1.3)
Psocoptera	13 (0.8)	17 (4.0)	11 (0.9)	6 (1.2)	2 (0.0)
Native	52 (5.0)	31 (6.8)	27 (0.8)	21 (3.3)	5 (1.3)
Endemic	16 (3.6)	11 (1.9)	13 (0.8)	24 (1.7)	13 (2.0)
Introduced	41 (2.1)	39 (6.8)	24 (1.9)	10 (1.4)	10 (1.2)
Araneae ind.	6 (1.5)	5 (1.4)	8 (1.2)	13 (2.1)	8 (2.0)
Coleoptera ind.	12 (1.4)	7 (1.2)	7 (0.9)	8 (0.9)	1 (0.0)
Hemiptera ind.	26 (2.6)	11 (2.1)	11 (0.9)	12 (2.1)	5 (0.7)
Psocoptera ind.	10 (0.9)	12 (3.1)	8 (0.8)	4 (1.3)	2 (0.0)

c) Transect mean richness q1

Species group	Elevation [m a.s.l.]				
	**46**	**231**	**404**	**693**	**930**
All	37 (6.7)	18 (14.2)	28 (1.3)	18 (7.1)	12 (1.2)
Araneae	3 (1.4)	4 (1.3)	4 (1.0)	6 (3.8)	4 (1.7)
Coleoptera	8 (1.8)	6 (4.9)	6 (0.6)	3 (0.5)	2 (0.6)

Hemiptera	11 (2.8)	4 (3.4)	6 (0.5)	5 (2.2)	3 (2.0)
Psocoptera	7 (1.7)	4 (3.9)	6 (1.2)	1 (1.0)	2 (0.0)
Native	19 (6.2)	7 (6.2)	14 (1.0)	8 (3.5)	2 (1.5)
Endemic	6 (1.5)	3 (1.7)	8 (0.5)	8 (3.2)	8 (1.0)
Introduced	12 (3.6)	8 (6.6)	6 (2.0)	2 (1.4)	2 (1.0)
Araneae ind.	2 (1.0)	1 (1.0)	2 (0.8)	5 (2.6)	4 (1.2)
Coleoptera ind.	3 (1.0)	2 (1.7)	3 (1.0)	3 (0.5)	1 (0.0)
Hemiptera ind.	11 (2.9)	4 (2.4)	6 (0.0)	5 (2.2)	3 (1.5)
Psocoptera ind.	6 (1.0)	3 (3.1)	5 (1.0)	1 (0.8)	2 (0.0)

d) All sites observed richness

Species group	Elevation [m a.s.l.]				
	46	231	490	672	910
All	125 (6.9)	91 (6.2)	74 (5.5)	61 (4.8)	42 (4.0)
Araneae	15 (3.4)	15 (2.4)	19 (3.1)	15 (2.3)	12 (2.0)
Coleoptera	47 (4.7)	33 (3.8)	23 (2.9)	20 (2.8)	13 (2.6)
Hemiptera	30 (3.2)	19 (3.8)	13 (2.6)	12 (2.4)	9 (1.5)
Psocoptera	11 (0.7)	11 (0.9)	8 (0.8)	6 (0.6)	4 (0.1)
Native	55 (3.9)	32 (4.1)	27 (3.0)	24 (3.3)	14 (2.1)
Endemic	12 (0.7)	11 (1.4)	20 (3.0)	20 (3.5)	18 (1.8)
Introduced	56 (5.8)	47 (4.5)	28 (3.3)	18 (1.9)	15 (3.0)

e) All sites estimated total richness

Species group	Elevation [m a.s.l.]				
	46	231	490	672	910
All	174 (5.4)	134 (11.8)	103 (5.9)	83 (6.7)	59 (4.7)
Araneae	24 (3.3)	22 (2.7)	28 (3.3)	21 (2.4)	14 (1.5)
Coleoptera	68 (4.1)	50 (4.5)	35 (2.3)	28 (3.3)	21 (2.9)
Hemiptera	43 (3.5)	30 (5.2)	18 (2.0)	16 (2.0)	12 (1.6)
Psocoptera	12 (0.9)	13 (1.3)	10 (1.0)	7 (0.7)	4 (0.4)
Native	73 (3.5)	46 (5.1)	36 (2.8)	31 (3.1)	22 (2.6)
Endemic	14 (1.9)	15 (1.7)	24 (2.2)	24 (1.7)	20 (1.8)
Introduced	84 (3.4)	71 (7.5)	45 (3.9)	28 (3.7)	26 (3.2)

f) All sites mean richness q1

Species group	Elevation [m a.s.l.]				
	46	231	490	672	910
All	29 (7.7)	16 (8.7)	17 (6.5)	16 (7.7)	10 (3.8)
Araneae	2 (1.3)	2 (1.3)	3 (1.7)	5 (2.5)	4 (1.7)
Coleoptera	8 (2.2)	5 (2.6)	3 (2.1)	3 (2.4)	2 (1.3)
Hemiptera	8 (2.8)	4 (2.4)	5 (1.6)	4 (1.8)	2 (1.3)
Psocoptera	5 (2.5)	3 (2.4)	3 (2.0)	2 (1.5)	1 (0.8)
Native	15 (4.7)	7 (4.4)	8 (4.0)	5 (3.8)	2 (1.7)
Endemic	4 (1.5)	3 (1.2)	7 (2.4)	8 (3.1)	7 (2.4)
Introduced	10 (3.0)	6 (4.1)	3 (2.2)	2 (2.0)	1 (0.9)

g) Exponential Shannon index q2

Species group	Elevation [m a.s.l.]				
	46	231	404	693	930
All	4.0	11.2	22.0	7.2	5.0
Araneae	6.2	6.2	4.7	10.0	4.6
Coleoptera	15.9	6.1	5.0	4.3	3.4
Hemiptera	2.1	2.5	3.8	2.9	1.9
Psocoptera	6.4	5.9	5.4	3.4	1.5
Native	2.5	4.3	11.6	7.9	3.1
Endemic	3.4	2.5	6.1	3.5	3.8
Introduced	8.3	10.1	6.8	4.3	5.7

h) Inverse Simpson index q3

Species group	Elevation [m a.s.l.]				
	46	231	404	693	930
All	1.9	4.4	15.1	3.3	2.9
Araneae	5.8	4.8	3.0	8.8	3.8
Coleoptera	11.3	3.5	4.2	3.7	2.5
Hemiptera	1.5	1.6	2.5	2.1	1.6
Psocoptera	5.4	4.0	4.4	2.9	1.4
Native	1.6	2.2	8.3	5.5	2.6
Endemic	2.7	1.7	4.8	2.2	2.5
Introduced	5.0	5.1	4.0	3.5	5.4

i) Berger-Parker index q4

Species group	Elevation [m a.s.l.]				
	46	231	404	693	930
All	1.4	2.1	6.4	1.9	1.8
Araneae	4.3	3.3	1.9	7.4	2.7
Coleoptera	4.8	2.0	4.0	2.7	1.7
Hemiptera	1.2	1.3	1.7	1.5	1.3
Psocoptera	3.7	5.2	2.7	2.0	1.2
Native	1.3	1.5	3.8	3.0	1.8
Endemic	2.2	1.3	1.8	1.5	1.7
Introduced	3.0	2.4	2.2	2.6	3.5

Table S4 Alpha-diversity linear regression coefficients (Df = 3 in all cases) of observed, estimated total and mean species richness patterns for all species, four different orders and native, endemic and introduced species over; a) the five-site elevation transect, b) only indigenous species on the elevation transect, and c) the five elevation bands that reflect all 12 sites.

a) Transect sites

Species group		All species	Araneae	Coleoptera	Hemiptera	Psocoptera	Native	Endemic	Introduced
Observed richness	Intercept	74.2	8.9	21.9	18.1	12.7	34.7	9.3	29.7
	Slope	-0.056	0.0011	-0.021	-0.015	-0.011	-0.032	0.0051	-0.029
	F-test	28.43	0.069	34.30	6.95	52.93	17.18	0.77	39.15
	P-value	0.013	0.81	0.0099	0.078	0.0054	0.026	0.45	0.0082
Estimated total richness	Intercept	105.4	13.0	32.9	25.4	16.8	47.8	13.4	43.1
	Slope	-0.082	0.00078	-0.032	-0.022	-0.015	-0.045	0.0040	-0.040
	F-test	46.40	0.017	26.33	7.32	15.66	27.87	0.25	29.89
	P-value	0.0065	0.90	0.014	0.073	0.029	0.013	0.65	0.012
Mean richness	Intercept	32.8	3.1	7.8	9.0	6.7	16.9	4.4	11.4
	Slope	-0.022	0.0019	-0.0062	-0.0067	-0.0058	-0.015	0.0045	-0.012
	F-test	5.13	2.09	71.02	3.59	5.46	5.34	2.34	34.82
	P-value	0.11	0.24	0.0035	0.15	0.10	0.10	0.22	0.010

b) Transect sites, indigenous species per order

Species group		Araneae	Coleoptera	Hemiptera	Psocoptera
Observed richness	Intercept	3.3	7.7	15.5	9.2
	Slope	0.0050	-0.0058	-0.012	-0.008
	F-test	2.24	5.32	5.25	35.18
	P-value	0.23	0.10	0.11	0.0096
Estimated total richness	Intercept	5.4	11.0	20.9	11.9
	Slope	0.0056	-0.0092	-0.018	-0.010
	F-test	2.09	8.12	5.30	15.90
	P-value	0.24	0.065	0.105	0.028
Mean richness	Intercept	0.9	2.9	8.5	5.3
	Slope	0.0035	-0.0010	-0.0064	-0.0043
	F-test	5.91	0.44	3.08	3.47
	P-value	0.093	0.56	0.18	0.16

c) All 12 sites

Species group		All species	Araneae	Coleoptera	Hemiptera	Psocoptera	Native	Endemic	Introduced
Observed richness	Intercept	120.8	16.3	44.5	27.4	12.1	49.7	11.5	57.0
	Slope	-0.090	-0.0025	-0.037	-0.023	-0.0088	-0.041	0.010	-0.051
	F-test	67.44	0.34	41.91	22.03	65.60	18.35	4.20	61.70
	P-value	0.0038	0.60	0.0075	0.018	0.0039	0.023	0.13	0.0043
Estimated total richness	Intercept	171.3	26.2	65.7	40.6	13.8	66.5	14.5	85.4
	Slope	-0.13	-0.0092	-0.054	-0.036	-0.010	-0.053	0.010	-0.074
	F-test	116.70	1.84	52.93	30.77	28.73	22.39	3.29	53.65
	P-value	0.0017	0.27	0.0054	0.012	0.013	0.018	0.17	0.0053
Mean richness	Intercept	25.9	1.6	6.8	6.9	5.0	13.0	3.4	9.3
	Slope	-0.018	0.0032	-0.0061	-0.0050	-0.0047	-0.012	0.0052	-0.010
	F-test	7.83	13.61	12.22	7.97	19.41	11.15	4.76	33.47

**P-value**	0.068	0.035	0.040	0.067	0.022	0.044	0.12	0.010

Table S5 Relative importance of the alpha-diversity (i.e. mean observed richness per sample) and beta-diversity components of the total observed species richness along the elevation transect for all species, four different orders and native, endemic and introduced species. Total beta-diversity is derived as the difference between total (gamma) diversity and alpha-diversity, and is the dominant component of total diversity in each group, except for Psocoptera and endemic species (column 4).

**Dominance of alpha- or beta-diversity**				
**Species group**	**Observed richness**	**Mean richness**	**Beta-diversity**	**% Beta-diversity**
All species	134	48	86	64
Araneae	33	9	24	73
Coleoptera	41	12	29	71
Hemiptera	28	11	17	61
Psocoptera	13	7	6	46
Native	53	20	33	62
Endemic	23	12	11	48
Introduced	57	16	41	72

Table S6 Beta-diversity measures over the five-site elevation transect for all species, four different orders and native, endemic and introduced species over adjacent sites (columns 1-4) and the remaining pairs of sites at larger elevational distances (column 5-10): a) Incidence-based and b) abundance-based estimates of total beta-diversity and its replacement and richness components, all based on Jaccard dissimilarity indexes.

a) Incidence-based beta-diversity

**Site A [m a.s.l.]**	**46**	**231**	**404**	**693**	**46**	**231**	**404**	**46**	**231**	**46**
**Site B [m. a.s.l.]**	**231**	**404**	**693**	**930**	**404**	**693**	**930**	**693**	**930**	**930**
**Distance [m a.s.l.]**	**185**	**173**	**289**	**237**	**358**	**462**	**526**	**647**	**699**	**884**
**Species group**	**Total beta-diversity**									
All species	0.70	0.65	0.67	0.70	0.67	0.86	0.89	0.82	0.89	0.90
Araneae	0.87	0.95	0.79	0.67	0.87	1.00	0.94	0.95	1.00	1.00
Coleoptera	0.79	0.67	0.69	0.90	0.83	0.90	1.00	0.96	0.95	0.96
Hemiptera	0.67	0.57	0.64	0.60	0.61	0.73	0.75	0.71	0.75	0.83
Psocoptera	0.31	0.18	0.56	0.50	0.18	0.64	0.78	0.64	0.82	0.82
Native	0.68	0.65	0.64	0.75	0.67	0.84	0.92	0.80	0.91	0.92
Endemic	0.36	0.50	0.47	0.47	0.53	0.75	0.71	0.68	0.71	0.71
Introduced	0.80	0.72	0.90	1.00	0.71	0.97	1.00	0.94	0.97	0.97
**Species group**	**Species replacement**									
All species	0.44	0.61	0.54	0.30	0.35	0.72	0.44	0.44	0.42	0.26
Araneae	0.67	0.95	0.63	0.27	0.67	0.87	0.75	0.63	0.82	1.00
Coleoptera	0.61	0.38	0.31	0.80	0.41	0.38	0.63	0.36	0.38	0.30
Hemiptera	0.17	0.57	0.57	0.20	0.09	0.67	0.33	0.17	0.33	0.09
Psocoptera	0.31	0.00	0.00	0.00	0.00	0.00	0.00	0.00	0.00	0.00
Native	0.27	0.58	0.43	0.00	0.31	0.71	0.17	0.31	0.18	0.05
Endemic	0.00	0.17	0.11	0.11	0.53	0.20	0.71	0.36	0.43	0.71
Introduced	0.71	0.44	0.40	1.00	0.34	0.33	0.55	0.24	0.33	0.29
**Species group**	**Species richness**									
All species	0.27	0.04	0.13	0.40	0.31	0.15	0.44	0.39	0.47	0.64
Araneae	0.20	0.00	0.16	0.40	0.20	0.13	0.19	0.32	0.18	0.00
Coleoptera	0.18	0.29	0.38	0.10	0.41	0.52	0.38	0.61	0.57	0.67
Hemiptera	0.50	0.00	0.07	0.40	0.52	0.07	0.42	0.54	0.42	0.74
Psocoptera	0.00	0.18	0.56	0.50	0.18	0.64	0.78	0.64	0.82	0.82
Native	0.41	0.06	0.21	0.75	0.36	0.13	0.75	0.49	0.73	0.87
Endemic	0.36	0.33	0.37	0.37	0.00	0.55	0.00	0.32	0.29	0.00
Introduced	0.09	0.28	0.50	0.00	0.37	0.63	0.45	0.70	0.63	0.68

b) Abundance-based beta-diversity

**Site A [m a.s.l.]**	**46**	**231**	**404**	**693**	**46**	**231**	**404**	**46**	**231**	**46**
**Site B [m. a.s.l.]**	**231**	**404**	**693**	**930**	**404**	**693**	**930**	**693**	**930**	**930**
**Distance [m a.s.l.]**	**185**	**173**	**289**	**237**	**358**	**462**	**526**	**647**	**699**	**884**
**Species group**	**Total beta-diversity**									
All species	0.95	0.88	0.84	0.77	0.95	0.96	0.80	0.98	0.95	0.97
Araneae	0.94	0.87	0.94	0.70	0.89	1.00	0.99	0.99	1.00	1.00
Coleoptera	0.86	0.88	0.80	0.98	0.91	0.96	1.00	0.97	0.99	0.99
Hemiptera	0.96	0.90	0.80	0.74	0.98	0.96	0.70	0.98	0.95	0.97
Psocoptera	0.86	0.83	0.96	0.94	0.60	0.88	0.74	0.97	0.90	0.96

Native	0.96	0.88	0.87	0.95	0.97	0.97	0.96	1.00	0.96	1.00
Endemic	0.90	0.87	0.81	0.73	0.68	0.94	0.68	0.83	0.93	0.71
Introduced	0.94	0.90	0.97	1.00	0.92	0.98	1.00	0.99	0.99	1.00
**Species group**	**Species replacement**									
All species	0.04	0.71	0.77	0.48	0.08	0.73	0.60	0.15	0.93	0.07
Araneae	0.60	0.76	0.82	0.49	0.44	0.80	0.94	0.49	0.96	0.64
Coleoptera	0.58	0.78	0.19	0.38	0.56	0.51	0.16	0.74	0.17	0.26
Hemiptera	0.01	0.63	0.14	0.38	0.01	0.58	0.29	0.13	0.86	0.05
Psocoptera	0.14	0.20	0.00	0.07	0.41	0.00	0.14	0.00	0.81	0.37
Native	0.02	0.83	0.43	0.19	0.04	0.53	0.06	0.03	0.05	0.00
Endemic	0.00	0.01	0.38	0.53	0.50	0.01	0.39	0.55	0.03	0.59
Introduced	0.37	0.69	0.32	0.56	0.20	0.24	0.16	0.07	0.10	0.03
**Species group**	**Species richness**									
All species	0.92	0.17	0.08	0.29	0.87	0.23	0.20	0.83	0.02	0.90
Araneae	0.34	0.10	0.12	0.21	0.44	0.20	0.05	0.50	0.04	0.36
Coleoptera	0.28	0.10	0.61	0.60	0.35	0.45	0.84	0.22	0.82	0.72
Hemiptera	0.95	0.26	0.66	0.36	0.97	0.38	0.41	0.84	0.09	0.93
Psocoptera	0.71	0.62	0.96	0.87	0.19	0.88	0.59	0.97	0.09	0.59
Native	0.94	0.05	0.44	0.76	0.94	0.44	0.90	0.96	0.91	1.00
Endemic	0.90	0.86	0.43	0.20	0.18	0.93	0.29	0.28	0.90	0.11
Introduced	0.58	0.20	0.65	0.44	0.72	0.74	0.84	0.92	0.89	0.97

Table S7 Multiple regression on distance matrices (MRM) coefficients of total beta-diversity and its replacement and richness components for all species, four different orders and native, endemic and introduced species, over the distance between sites, for; a) incidence-based and b) abundance-based beta-diversity estimates, all based on Jaccard dissimilarity indexes.

a) Incidence-based beta-diversity

Species group		All species	Araneae	Coleoptera	Hemiptera	Psocoptera	Native	Endemic	Introduced
**Total**	**Intercept**	0.61	0.78	0.70	0.55	0.18	0.60	0.38	0.78
	**Slope**	3.80E-04	2.84E-04	3.72E-04	2.99E-04	8.21E-04	4.05E-04	4.67E-04	2.60E-04
	**F-test**	23.65	5.36	10.94	29.17	14.70	20.01	17.48	3.66
	**P-value**	0.029	0.064	0.020	0.023	0.033	0.011	0.017	0.077
**Replace-ment**	**Intercept**	0.54	0.55	0.59	0.44	0.10	0.45	-0.27	0.72
	**Slope**	-1.95E-04	3.85E-04	-3.07E-04	-2.79E-04	-1.54E-04	-3.23E-04	8.04E-04	-5.68E-04
	**F-test**	1.03	1.98	2.00	0.86	1.36	1.11	10.75	4.22
	**P-value**	0.40	0.18	0.20	0.47	0.30	0.36	0.020	0.10
**Richness**	**Intercept**	0.07	0.22	0.11	0.11	0.08	0.15	0.41	0.64
	**Slope**	5.75E-04	-1.02E-04	6.78E-04	5.77E-04	9.75E-04	7.29E-04	-3.37E-04	8.28E-04
	**F-test**	11.22	0.33	27.74	3.92	14.36	4.71	1.74	15.12
	**P-value**	0.020	0.55	0.032	0.055	0.029	0.011	0.31	0.021

b) Abundance-based beta-diversity

Species group		All species	Araneae	Coleoptera	Hemiptera	Psocoptera	Native	Endemic	Introduced
**Total**	**Intercept**	0.84	0.82	0.85	0.83	0.80	0.90	0.83	0.93
	**Slope**	1.46E-04	2.48E-04	1.88E-04	1.38E-04	1.33E-04	1.18E-05	-5.90E-05	9.59E-05
	**F-test**	2.14	5.34	6.16	0.86	0.62	6.19	0.16	5.00
	**P-value**	0.132	0.031	0.016	0.461	0.471	0.100	0.757	0.020
**Replace-ment**	**Intercept**	0.52	0.63	0.60	0.29	0.01	0.48	0.14	0.06
	**Slope**	-1.45E-04	1.43E-04	-3.76E-04	4.23E-05	4.52E-04	-5.91E-04	3.64E-04	-7.66E-04
	**F-test**	0.08	0.27	1.38	0.01	1.78	2.66	1.06	22.03
	**P-value**	0.88	0.60	0.34	0.88	0.25	0.19	0.369	0.01
**Richness**	**Intercept**	0.32	0.19	0.25	0.54	0.79	0.42	0.70	0.31
	**Slope**	2.90E-04	1.05E-04	5.63E-04	9.61E-05	-3.20E-04	7.09E-04	-4.23E-04	8.62E-04
	**F-test**	0.28	0.19	3.03	0.04	0.54	3.25	0.76	25.77
	**P-value**	0.786	0.70	0.100	0.915	0.508	0.087	0.49	0.018

Table S8 Relative dominance of the replacement or richness components of beta-diversity for all species, four different orders and native, endemic and introduced species, for both incidence- and abundance-based beta-diversity estimates, tested by Wilcoxon signed-rank tests. Replacement is dominant for Araneae whereas richness is dominant for Psocoptera and for native and endemic species in abundance-based beta-diversity estimates.

Replacement or richness dominance

**Species group**	**Incidence**		**Abundance**	
	**V**	**P-value**	**V**	**P-value**
All species	33	0.24	26	0.92
Araneae	54	0.0080	44	0.013
Coleoptera	25	0.86	24	0.77
Hemiptera	24	0.76	15	0.23
Psocoptera	3	0.014	7	0.037
Native	16	0.28	6	0.027
Endemic	30	0.84	20	0.49
Introduced	25	0.84	5	0.020

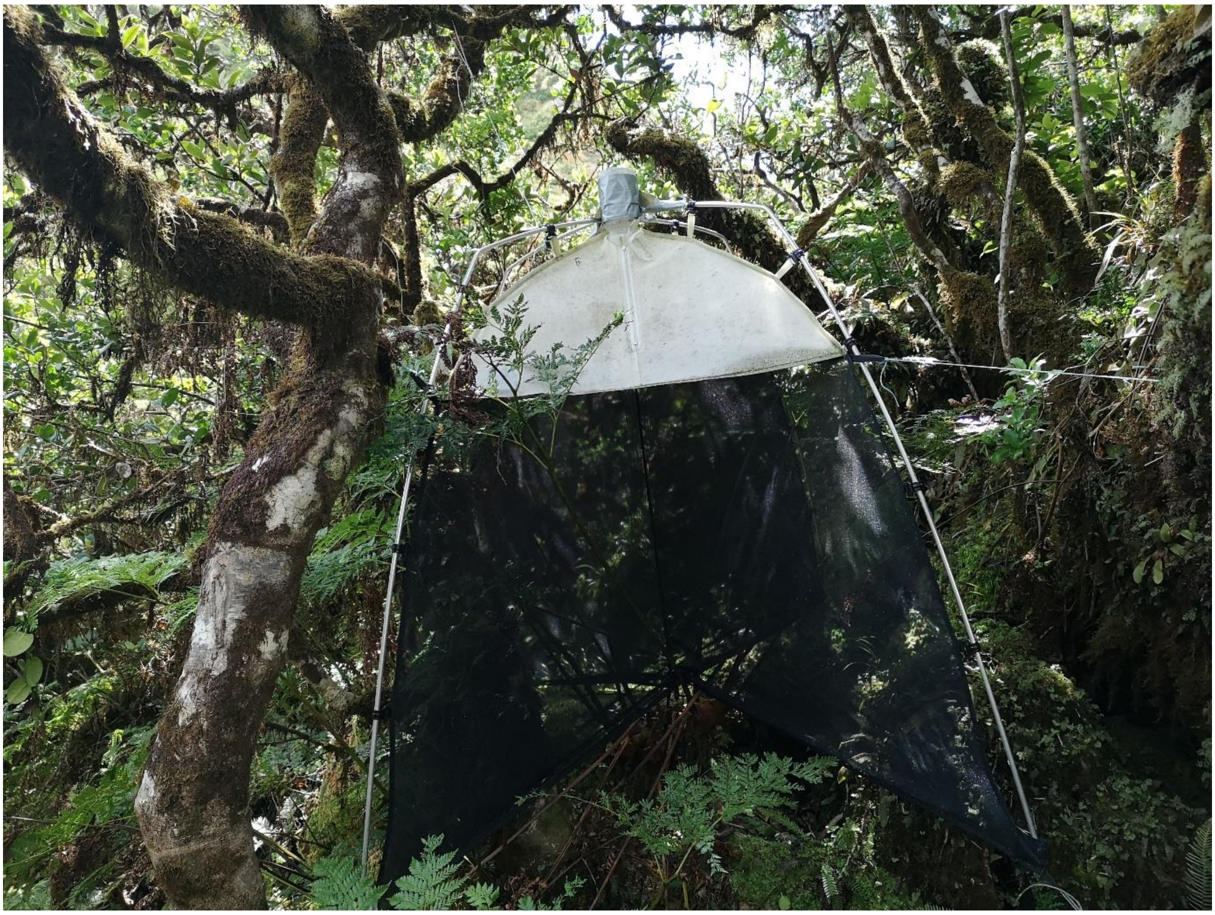

Figure S1 Image of a SLAM (Sea, Land, and Air Malaise) trap (Credit: Paulo A.V. Borges).

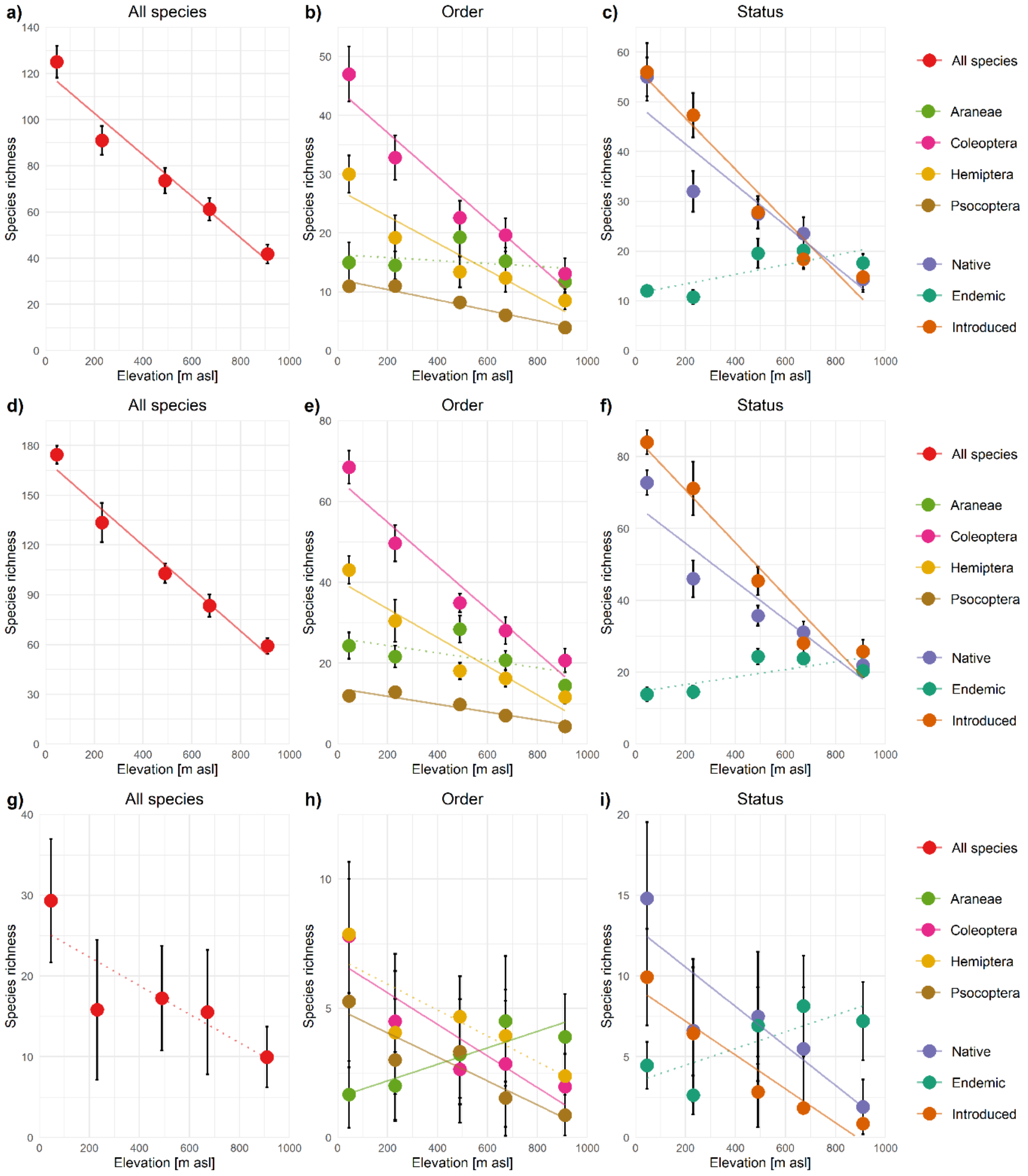

Figure S2 Species richness patterns for all species, four different orders and native, endemic and introduced species over the five elevation bands (represented by mean elevation) that reflect all 12 sampling sites on Terceira island (records of each season from autumn 2014 to summer 2018, rarified to a common sampling level of 15 samples per band): a) – c) Observed species richness, d) – f) estimated total (Jack1) species richness, and g) – i) mean richness per sample (q1). The bars indicate the standard deviations of each estimate. Linear trendlines are shown as solid lines (p-values < 0.05) or indicated with dotted lines ($p > 0.05$).

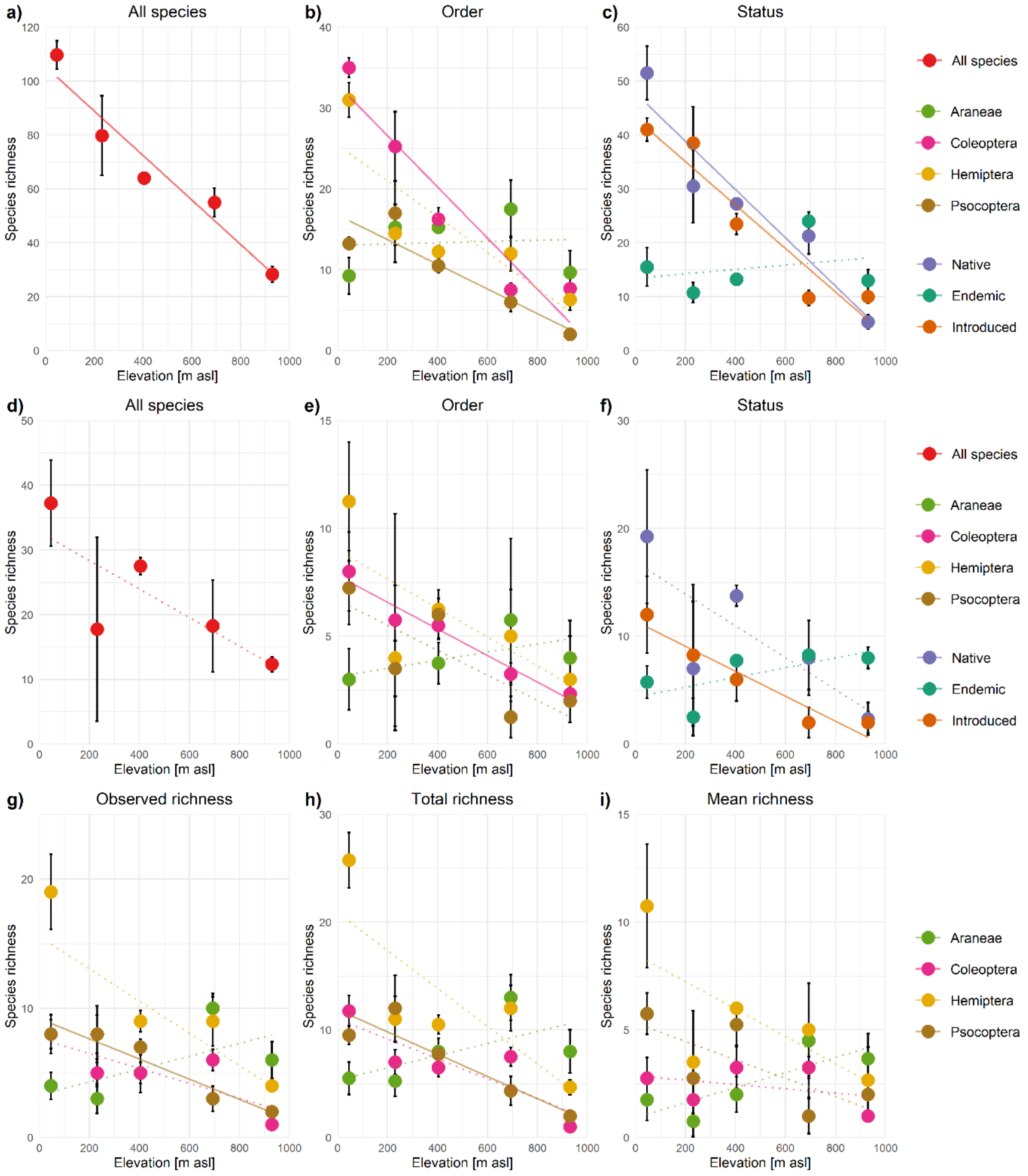

Figure S3 Species richness patterns for all species, four different orders and native, endemic and introduced species over the five sites of the elevation transect (summer records of the years 2015-2018): a) – c) Estimated total (Jack1) species richness, d) – f) mean richness per sample (*q*1), and g) – i) observed, estimated total (Jack1) and mean species richness per order for indigenous species only. The bars indicate the standard deviations of each estimate. Linear trendlines are shown as solid lines (p-values < 0.05) or indicated with dotted lines ($p > 0.05$).

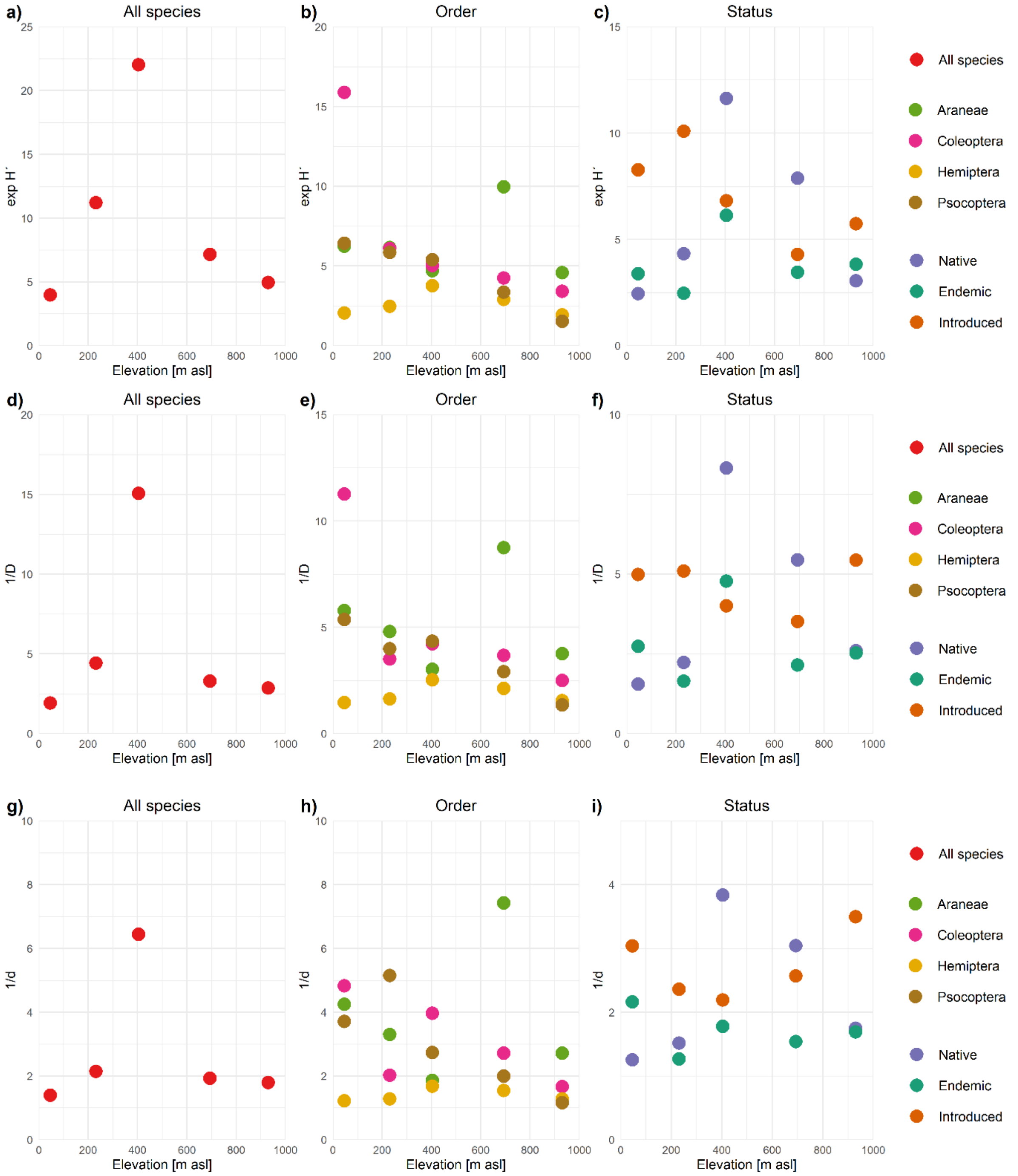

Figure S4 Hill numbers, indicating eveness in species distributions, for all species, four different orders and native, endemic and introduced species over the five sites of the elevation transect (summer records of the years 2015-2018): a) – c) Exponential Shannon index q2 (exp H'), d) – f) inverse Simpson index q3 (1/D), and g) – i) Berger-Parker index q4 (1/d).

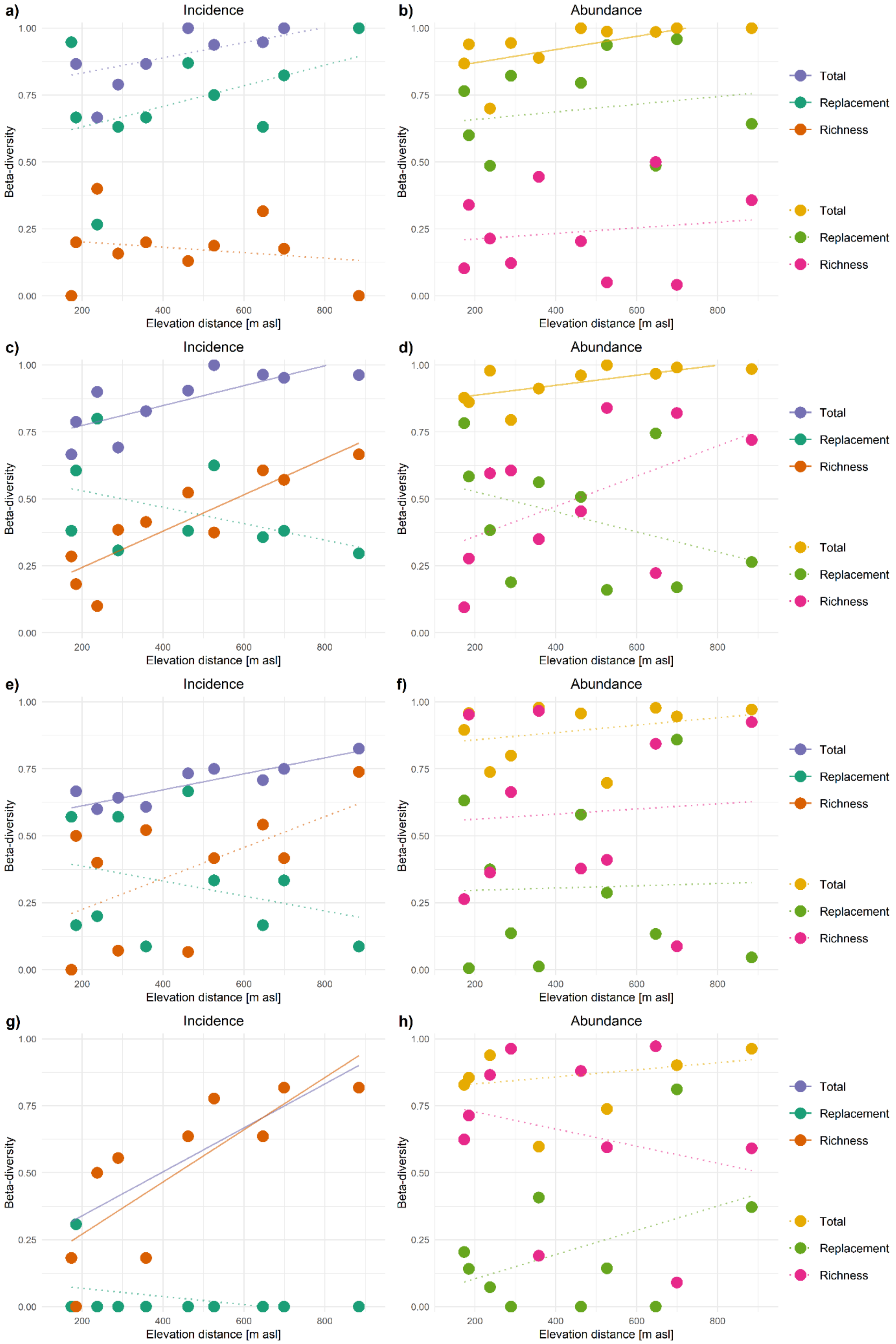

a)
Incidence
b)
Abundance
c)
Incidence
d)
Abundance
e)
Incidence
f)
Abundance
g)
Incidence
h)
Abundance
Beta-diversity
1.00
0.75
0.50
0.25
0.00
200
400
600
800
Elevation distance [m asl]
Total
Replacement
Richness
Total
Replacement
Richness

Previous page: Figure S5 Patterns of total beta-diversity and its replacement and richness components over the elevational distance between sites on the five-site elevation transect, providing incidence-based (left column) and abundance-based (right column) beta-diversity estimates for: a) – b) Araneae, c) – d) Coleoptera, e) – f) Hemiptera, g) – h) Psocoptera. Linear trendlines are shown as solid lines (*p*-values < 0.05) or indicated with dotted lines (*p* > 0.05).

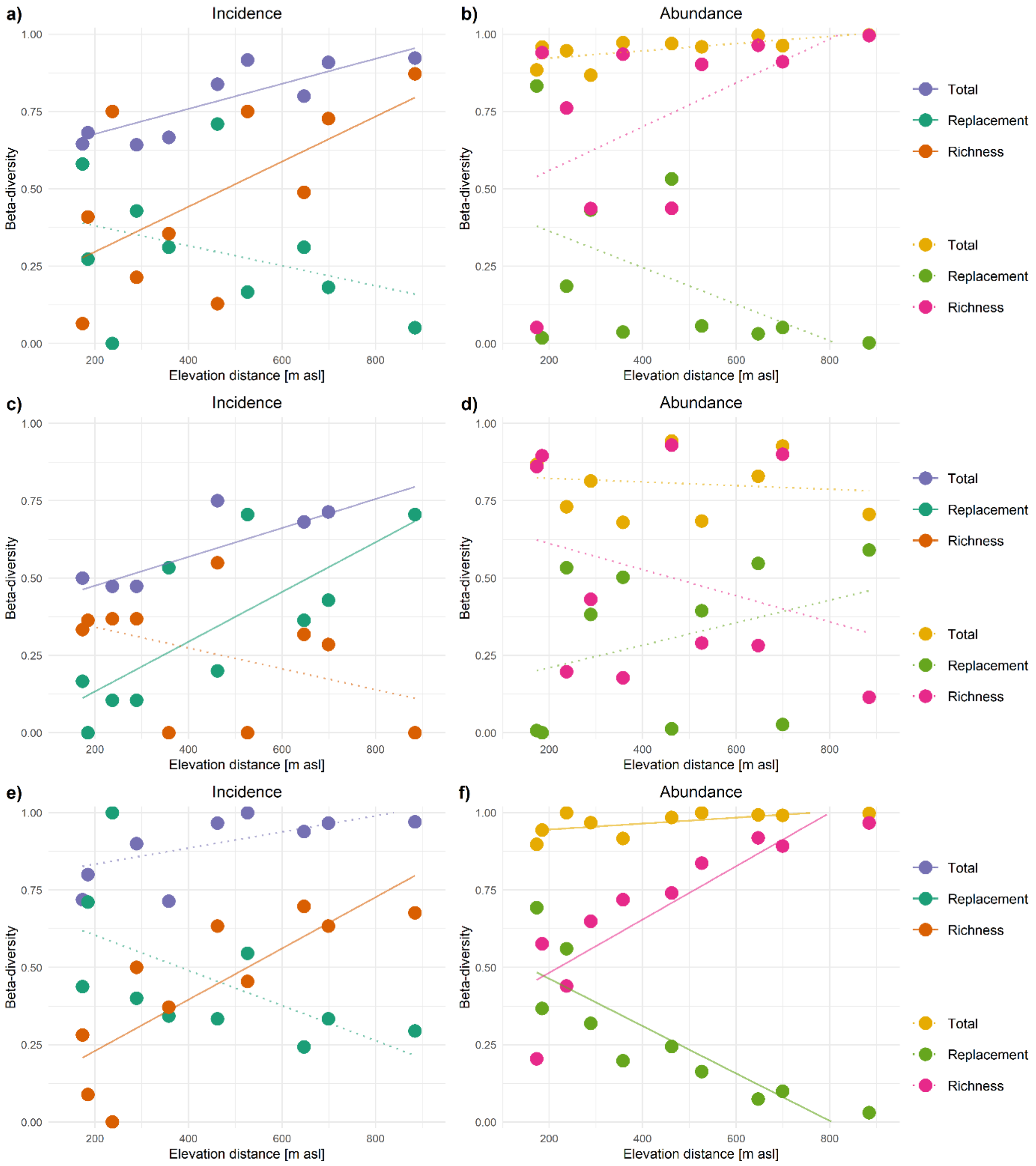

Figure S6 Patterns of total beta-diversity and its replacement and richness components over the elevational distance between sites on the five-site elevation transect, providing incidence-based (left column) and abundance-based (right column) beta-diversity estimates for: a) – b) Native species, c) – d) endemic species, e) – f) introduced species. Linear trendlines are shown as solid lines (*p*-values < 0.05) or indicated with dotted lines (*p* > 0.05).
